# eDNA-Based Survey of Fish Species in Water Bodies Using Loop-Mediated Isothermal Amplification (LAMP) for Application of Developing Automatic Sampler

**DOI:** 10.3390/mps7060085

**Published:** 2024-10-23

**Authors:** Nivedhitha Jothinarayanan, Chau Ha Pham, Frank Karlsen, Lars Eric Roseng

**Affiliations:** Department of Microsystems, University of South-Eastern Norway, 3184 Raveien, Norway; nivedhitha.narayanan@usn.no (N.J.); chau.pham@usn.no (C.H.P.); frank.karlsen@usn.no (F.K.)

**Keywords:** eDNA investigation, LAMP, Northern Pike, European Eel, Atlantic Salmon, housekeeping gene, eDNA analysis, lake

## Abstract

The monitoring of species in a habitat is important to ensure biological diversity. Environmental DNA (eDNA) can infer the presence–absence of species and enable rapid action to avoid threatening factors in ecosystems in the case of non-indigenous species. Loop-mediated isothermal amplification (LAMP) assays for molecular amplification are rapidly gaining popularity in species detection, but LAMP remains an underutilized method for eDNA-based monitoring practices. The most effective combination for successful species monitoring may be the collection of eDNA or biological traces collected by nanofiltration followed by LAMP-based species detection initiatives. Here, we used LAMP analysis to detect the eDNA of *Esox lucius* (Northern Pike), *Anguilla anguilla* (European Eel), and *Salmo salar* (Atlantic Salmon) in Borre Lake and in the Drammen River. The selection of species is based on the categories of regionally invasive species, endangered species, and species of least concern. Two target genes were considered for each species and LAMP primers were designed. Our study showed that LAMP is an effective tool for discovering specific fish eDNA (analysis) to maintain aquatic ecosystems.

## 1. Introduction

Environmental DNA (eDNA) analysis has revolutionized the field of biodiversity monitoring by providing a minimally disruptive way to detect genetic material that organisms have released into their environment. eDNA represents all kinds of biological traces that can be released from any biological organism. This genetic material makes it possible to identify the species that live in a particular ecosystem, without the need for direct observation [1,2]. The majority of eDNA studies have concentrated on species-specific detection that includes the identification of invasive and vulnerable species [3]. The use of eDNA sampling and analysis can be particularly beneficial for following the spread of imported species, identifying them, and determining their pathways, as well as keeping an eye on any potential survivors as part of ongoing eradication efforts. Even a relatively small number of invasive species can cause significant changes in freshwater ecosystems and affect the integrity of these ecosystems.

Among various molecular techniques, loop-mediated isothermal amplification (LAMP) [4] has gained prominence due to its rapid amplification rate under isothermal conditions, making it an ideal tool for eDNA detection. The specificity and sensitivity of target detection is greatly facilitated by LAMP, which functions efficiently under isothermal conditions due to the strand displacement activity of specific Bst (*Bacillus stearothermophilus*) DNA polymerase enzymes [5], and the introduction of loop primers has further improved the reaction rate [6]. DNA amplification using the LAMP method is important because this method is isothermal, cost-effective, rapid, and can be performed by operators with little training if used in an automatic system inside a lab-on-a-chip platform with freeze-dried reaction reagents [7,8,9].

According to the IUCN (International Union for Conservation of Nature), some species are critically endangered and fall under the red list category [10]. The species included in this eDNA survey were selected based on the red list category, regional alien status, and status of least concern, and the order is as follows: *Anguilla anguilla* (European Eel), *Esox lucius* (Northern Pike—alien in the southeastern parts of Norway), and *Salmo salar* (Atlantic Salmon). The Northern Pike mostly lives in freshwater, such as in lakes and rivers, and it is a predator fish for other small fishes [11]. The other two fishes follow a life cycle spanning from the river to the ocean and back to the river. Traditional PCR techniques have been widely applied in field investigations where samples are obtained for laboratory analysis, in the examination of aquatic species, and in eDNA determination [12,13]. In recent times, LAMP has become much more suitable because of its robustness in clinical diagnosis and field sample analysis, where resources are limited [14,15].

Two housekeeping genes were targeted for each of the fish species. All gene sequences were obtained from the National Center for Biotechnology (NCBI)’s database, and the selection criterion was that the biomarkers should have less cross-homology to ensure specific species’ identification. The most common biomarker used to characterize fish eDNA is the mitochondrial Cytochrome B (Cyt B) gene [16]. The reason for choosing the Cyt B gene is that the copy number of mitochondrial DNA will be high in the environmental sample [17]. Thus, the Cyt B gene was chosen for Northern Pike and Atlantic Salmon, and in addition, the genes coding for Glyceraldehyde-3-Phosphate Dehydrogenase (GAPDH) and Elongation factor (EF) were also chosen for these species. For the European Eel, the genes that code for Polymerase III polypeptide (PIII) and RNA polymerase I subunit H (PIH) were selected as biomarkers.

Nanofiltration (NF) is very important to ensure optimal and representative collection of all important biological materials in water, and several studies have proven the effectiveness of the nanofiltration membranes [18,19]. Nanofiltration is also emerging as an important technique within the field of separation of biological compounds based on its durability, efficiency, ease of use, and low cost [18]. The main advantage of using a hydrophilic NF system is the ability to concentrate enough biological traces and bioactive compounds that are likely to represent all eDNA or eRNA in the selected sample [19].

An advantage of LAMP, compared to traditional PCR, is that the LAMP reaction takes place isothermally at a temperature of 65 °C. In the LAMP reaction, there is no requirement for a denaturation step, where the temperature must be raised cyclically to 95 °C. An automatic system for on-site, real-time eDNA detection in the field is therefore easier to design if LAMP amplification is chosen instead of PCR.

In this work, we have designed different LAMP primers for fish species such as European Eel, Atlantic Salmon, and Northern Pike. The Northern Pike primers have been previously validated in our experiments [8]. Nanofiltration of water from Borre Lake and the Drammen River in Norway has been carried out to detect eDNA of the targeted species in these water bodies using the LAMP technique. The eDNA collection and analysis work was performed to test their possible use in the development of an automatic lab-on-a-chip system for on-site, real-time environmental monitoring of lakes and rivers.

## 2. Materials and Methods

**Figure 1:**
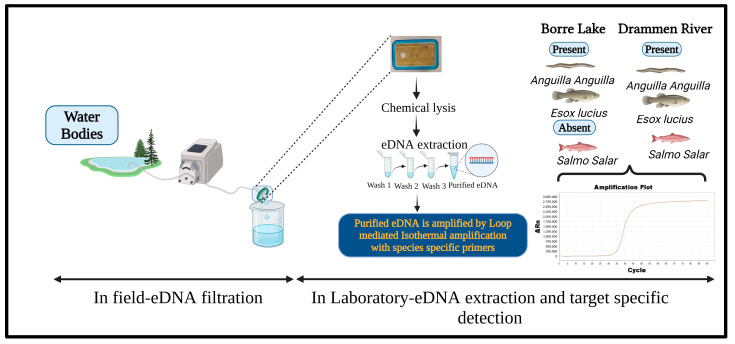
Schematic of work carried out in the entire eDNA survey of water samples collected from Borre Lake and the Drammen River. Lake/river water was filtered directly in the field and brought into the laboratory for eDNA extraction and detection, as seen in two parts.

A volume of 800 mL of water was filtered, and eDNA samples were collected on 0.2 µm nanofilters (Product Code: 17144321, Cytiva AcroCap Filter, Marlborough, MA, USA) from Borre Lake (located: 59°41′63.8″ N, 10°42′97.0″ E) at a depth of ~25 cm on the lakeside and the Drammen River (located: 59°77′29.8″ N, 9°91′34.3″ E) at a depth of ~20 cm at the riverside, both in Norway. The filters were stored on ice and brought into the laboratory for further processing. The entire process for eDNA extraction from the filters was performed within 7 days (at one location) to avoid the loss of eDNA and is categorized into two main steps, as described in Figure 1. To the filter was added 3 mL of lysis buffer and incubated for 5 min at 90 °C. The liquid was then ejected out to perform binding and washing of eDNA with silica beads. The eDNA extraction was performed using the Boom extraction method with the NUCLISENS MINIMAG kit (BioMerieux, Lyon, France). The kit protocol insists on three washes. Finally, the concentrated eDNA was eluted from the beads and used as a template in the LAMP reaction. The warm start LAMP kit (New England Biolabs, Ipswich, MA, USA) was used for the reaction setup, and the kit consisted of a warm start LAMP master mix and a fluorescent dye. The reaction was carried out in a 25 µL volume, consisting of 23 µL of master mix, primers, and fluorescent dye and 2 µL of template eDNA. The master mix contains a combination of dNTPs, *Bst* DNA polymerase, Tris HCl, KCl, Tween, (NH_4_)_2_SO_4_, Betaine, and MgSO_4_ in appropriate concentrations, as described by the warm start manual. The LAMP reaction was performed at 65 °C for 1 h and set with 80 cycles in Step OnePlus—Applied Biosystems (Thermo Fisher Scientific, Branchburg, NJ, USA). The LAMP primers for selected genes for each species were designed with certain criteria such as primer length, GC content, and loop primer temperature. The primers were designed by LAMP designer software version 1.01 (PREMIER Biosoft, San Francisco, CA, USA) and then procured from Eurofins Genomics (Aarhus, Denmark). Primers are listed in Table 1. The absence of primers PIH and EF from the provided table suggests that these primers do not facilitate the amplification of the target DNA.

The procured primers were reconstituted based on the concentrations of 0.2 µM, 0.4 µM, and 1.6 µM for F3/B3, Loop F/B, and FIP/BIP, respectively. Our previous study has determined the specificity and sensitivity of the designed Northern Pike LAMP primers using a tissue sample as template DNA [8], and the European Eel and Atlantic Salmon LAMP primers are novel in this investigation. In our previous studies, the LAMP primers demonstrated better sensitivity and specificity for Northern Pike [8], so we decided to expand this approach to other species using different genes.

## 3. Results and Discussion

**Figure 2:**
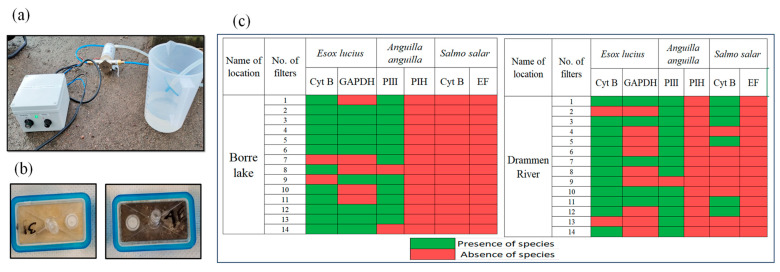
(**a**) On-site filtration setup (at the water bodies). (**b**) Appearance of some filter units after filtration of 800 mL water. (**c**) Plot of the presence or absence of target species from processed filters. Genes of each target are as follows: *Esox lucius*—Cytochrome B (Cyt B), Glyceraldehyde-3-Phosphate Dehydrogenase (GAPDH); *Anguilla anguilla*—Polymerase III polypeptide (PIII), RNA polymerase I subunit H (PIH); *Salmo salar*—Cytochrome B (Cyt B), Elongation Factor (EF).

Currently, there are no standard guidelines for eDNA technology for environmental monitoring. The methodology differs from laboratory to laboratory. The entire process from field to laboratory, carried out by us, is given schematically in Figure 1. Volumes of 800 mL of water from different locations (n = 3) of Borre Lake and the Drammen River were filtered, and the eDNA, together with particles in the water, was collected on different filters. An electronic peristaltic pump was used for the on-site freshwater filtration, and the setup is shown in Figure 2a. No negative control was taken at the time of sampling, but negative controls were included during the amplification of eDNA in the laboratory, and these were perfectly linear as a negative signal, which is not above the threshold value. The filtration volumes mentioned in the literature range from 15 mL to 6 L and even higher for eDNA collection [20,21]. However, filtering larger volumes is often challenging, due to sediment from the river or lake water clogging the filters. The volume of water that was filtered in our study was around 800 mL in each filter, and after this filtration, visible sediments were observed on the filters, as exhibited in Figure 2b. The filters chosen in our study had a pore size of 0.2 µm, with an area of 15 cm^2^, and the filters were made of hydrophilic polyether sulfone membranes and had been sterilized by gamma irradiation. The inlet of the filter was connected directly to the hose where water from the water body was pumped directly through the filter. The filtration was terminated after 800 mL of water had flowed through the filters. The filtration setup provides the same filtration condition for all samples. Sterile conditions were maintained, such as cleaning the apparatus with RNAse away solution, and the same method was followed during the laboratory experiments. All samples were collected between the spring and summer season (May to June 2022).

According to our results from different locations in Borre Lake and the Drammen River, Northern Pike is present in most of our sample locations. For the detection of Northern Pike, LAMP analysis was performed with two target genes, Cyt B and GAPDH, as shown in Figure 2c. Among the two European Eel primers, only one primer, the PIII LAMP primer, was amplifying the target, and the other PIH LAMP primers did not target the gene in the template. This was validated with the positive control, which is European Eel tissue. The absence of Atlantic Salmon in Borre Lake was indicated by our samples, and the possibility of Atlantic Salmon being present in Borre Lake is therefore limited. Furthermore, our results show a high probability of the presence of Northern Pike and European Eel in Borre Lake.

On the other hand, in the Drammen River, the analyses of our eDNA-filtered samples show the presence of all three species, but not at all sampling sites, as shown in Figure 2c. Another finding was that the samples for Northern Pike were more positive than those for Atlantic Salmon. However, there is not a clear connection between occurrence of eDNA and fish species abundance [22], and eDNA detection is linked only uncertainly to the size of the population of the target species. In this context, it is not possible to determine the population of the target species. Unfortunately, the PIH and EF biomarkers did not amplify in any of the eDNA samples or for the tissue sample positive control, indicating that there is an error related to the design of these LAMP primers. The chosen PIH and EF gene LAMP primers from our study are therefore not recommended, and they need to be further analyzed, using bioinformatic tools, in our future work. However, the results suggest that the LAMP primers from the Cyt B, GAPDH, and PIII genes can be recommended for new studies to identify Northern Pike, European Eel, and Atlantic Salmon. In some of the filter samples, where these positive biomarkers were tested, no amplification of the desired DNA was observed. These negative results can be due to various factors, such as the copy number, the gene of interest, levels of damage in the DNA, oxidative stress in the desired gene, and so on [23]. The original eDNA state can also be drastically changed by several factors such as pH, temperature, microbial activity, mineralogy, and microbial activity [24].

**Figure 3:**
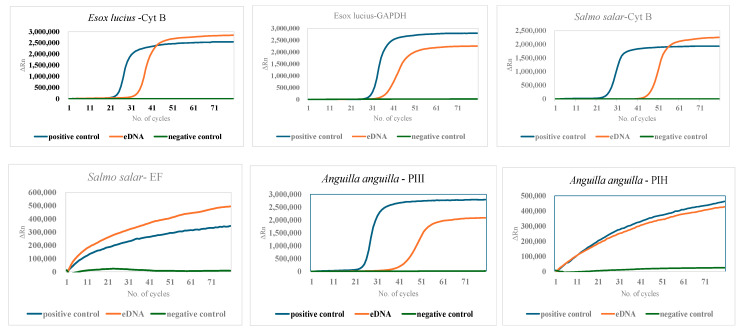
Amplification plots of eDNA collected from Borre Lake in filter number 3 and for the *Salmo salar* amplification from the Drammen River in filter number 5.

The eDNA obtained from the filters after the filtration process was amplified using the relevant primers, as depicted in Figure 3. The fluorescence values (ΔRn) of the reporter dye during the standard exponential amplification phase are shown for all the target species in Figure 3. Upon eDNA amplification, a distinct exponential phase similar to that of the positive control (tissue-extracted DNA) was observed. As expected, the negative control did not exhibit an exponential phase but showed a linear trend for all biomarkers and was given as negative control in Figure 3. It was noted earlier that the PIH and EF primers did not yield any amplification even in the positive control, as indicated in Figure 3.

**Figure 4:**
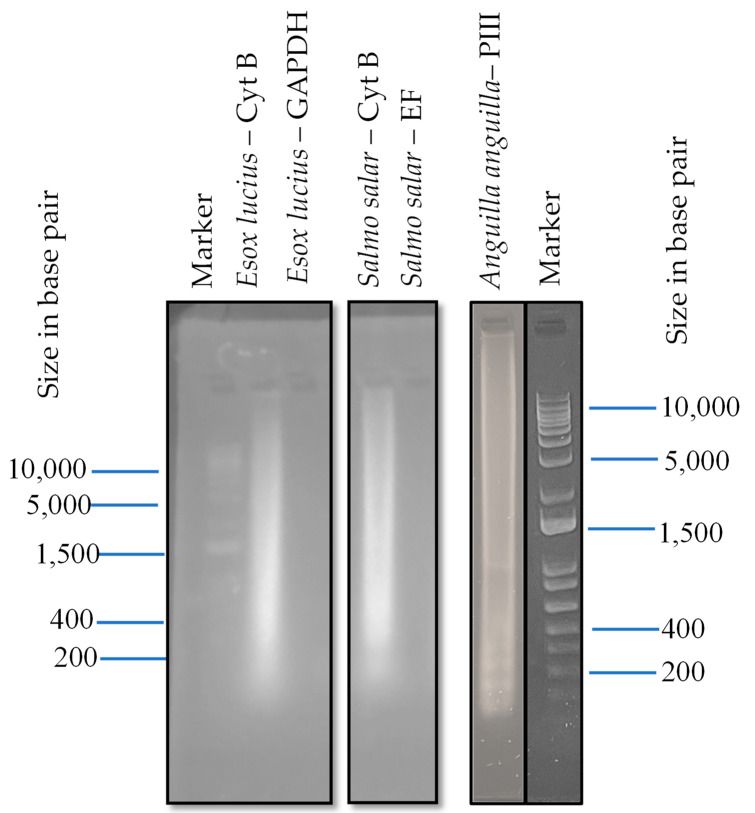
Results of 1% Agarose gel for LAMP product from Drammen River eDNA sample: filter number 9—*Esox lucius*; filter number 5—*Salmo salar*; Borre filter number 3—*Anguilla anguilla*.

Results from LAMP amplification of eDNA samples obtained from the Drammen River are presented in Figure 4. The LAMP products were analyzed using a 1 kb DNA ladder in which there is 10,000 to 200 base pairs. The DNA bands looked very weak and smeared possibly because of the inadequate sample volume or a lower gel percentage used. It was shown, though, that eDNA was present and amplification successful in filter 9 for *Esox lucius* (Cyt B) and filter 5 for *Salmo salar* (Cyt B), which were acquired from the Drammen River. No amplification, however, was observed for *Esox Lucius*—(GAPDH) or *Salmo salar* (EF), and these samples were also clear of any DNA bands. The Borre filter number 3 for *Anguilla anguilla* was seen as a smear with a slight band around 600 base pairs in a gel after amplification and compared with the 1 kb ladder in Figure 4.

This type of research can serve as an orientation to further develop current manual sampling and analysis techniques for the on-site, real-time monitoring of aquatic ecosystem in the field, where resources are limited. By standardizing the entire process for eDNA collection and analysis, autonomous systems for filtering water and detecting specific eDNA biomarkers can be established. The use of isothermal LAMP amplification in an automated system is a new investigative technique to detect high risk biosecurity environmental threats and will be useful in routine on-site surveillance programs conducted by biosecurity managers [25]. This study emphasizes aquatic eDNA monitoring using the rapid LAMP method supported by this eDNA-based survey study.

The use of LAMP technology in eDNA methods has a lot of potential for detecting DNA in the environment on the spot and in real time. This becomes even more exciting when we think about using this idea to create an automatic monitoring system. The ability of the isothermal LAMP amplification process is to bridge the gaps in current practices for on-site detection of DNA makes it an important tool for automated field-based applications.

This work focuses on how nanofiltration, eDNA research, housekeeping genes, and LAMP technology can work together to improve our ability to find and track = non-indigenous species that exist regionally in an aquatic environment, like the Northern Pike. A combination of these methods can increase the precision, efficiency, and usefulness of eDNA analysis for fisheries management and conservation of natural diversity. For eDNA-based detection studies, sensitivity is crucial because the signal can be significantly weakened due to the low density of copies of the biomarker. For optimal use, eDNA approaches require careful planning and consideration of several aspects.

To ensure that the desired target species are detected, enough filtered water is needed per sample, and the sampling plan and analysis techniques must be carefully selected based on the species of interest, the density and distribution of the targets, as well as the local environment [26]. The findings from this study were that eDNA could be captured from a sample volume of 800 mL after filtration through 0.2-micron-pore-size filters to detect target fish species such as European Eel, Northern Pike, and Atlantic Salmon in Borre Lake and the Drammen River. Furthermore, Cyt B, GAPDH, and PIII LAMP primers were recommended, whereas EF and PIH LAMP primers were not recommended for future studies when available in the database. In addition to presenting the positive results, we also report primers that were ineffective for the LAMP reaction in detecting species eDNA. This comprehensive approach provides valuable insight into both successful and unsuccessful primer designs for the research community. Furthermore, we will conduct an in-depth sensitivity analysis for every primer used in this study to assess their ability to detect a series of eDNA concentrations. This analysis will investigate the relationship between primer performance and eDNA concentration to determine the effective ranges of various primers. Through this systematic study, we aim to optimize our detection methods and enhance the precision and consistency of eDNA evaluation in the management of ecological monitoring. In many countries, a large amount of money and effort is spent to monitor invasive species. Consequently, eDNA-based LAMP technology in an autonomous system can be a sensitive, less time-consuming method and the best alternative to the conventional monitoring method.

## 4. Conclusions

In summary, the combination of nanofiltration to capture eDNA and isothermal LAMP technology has the potential to reform the detection and management of fish species in freshwater aquatic ecosystems. The findings indicate the effectiveness of the approach in identifying vulnerable and non-indigenous species, including European Eel and Northern Pike. As research in this field continues, an automated, on-site, real-time eDNA monitoring system could be developed to monitor and identify target species in real time, helping aquatic ecosystem conservation. Isothermal LAMP amplification for eDNA detection in freshwater ecosystems is a beneficial technology for aquatic environmental monitoring. This will help us better monitor aquatic ecosystems and ensure their sustainability.

## Figures and Tables

**Table 1:** shows the LAMP primer sequence for the selected species.

Name of the Species	Name of the Gene and Primer Sequence		Name of the Primers
	CYT B (Gene ID:NC_004593.1)	GAPDH (Gene ID: XM_010884975.4)	
*Esox Lucius*	TACACCACAGGGCTTGATA	CAGAGGACCAAGTT GTGTC	F3
	GCATGGGCTGTAACGATAA	CAAGTCGAGGGCTA GAGT	B3
	AGGGTGCCAATATCTTT GTGGTTCTCAGCCATCCTA CCTG	GCTTGACAAAGTGGTCGTTCAG ACATTCGCTCCTCCATCT	FIP
	AGTCGGCACAGCCTTA AGCCTGGTCGTCACCTA AGAGA	TACAGCAACCGCGTCATT GAGGTCGATTGGCTTTACTCC	BIP
	ATCAGCGTGTGATTGCCA	GCAATTCCAGCACCAGCATC	Loop F
	CCGAACTAAGCCAGCCAG	GATGGCTCACATGACCAC CAA	Loop B
	PIII (Gene ID:NC_049201.1)		
*Anguilla Anguilla*	GTGCTGTTTGCTGGGTAT		F3
	TGGAGGATAATGAGAACAGGA		B3
	CTGATGAGGTCGGTGATGGCATCGTAATTCGTGTCCAGAC		FIP
	CGAGTTGTCCTTGCTGGAAGAAA CTACAGCCTTCATTCAATCC		BIP
	ATTGGTGAAGGCCTCCTG		Loop F
	GCCATCAAAGACAAACAGGAAG		Loop B
	Cyt B (Gene ID:AF053591.1)		
Salmo Salar	TTCTGAGGAGCCACTGTAA		F3
	AGGATGTTAGGCCAAGTAGTA		B3
	GGAATAGGAAGTGGAAGGCGAAGCCCTTGTACAATGAATTTGAG		FIP
	GCTGCCACAGTACTCCATCTTCTATCGGCATCGGAGTTGA		BIP
	GGTGGCGTTGTCTACAGAA		Loop F
	GTCTAATAACCCAGCAGGCA		Loop B

## Data Availability

All the data supporting this study are available within the paper.
